# The Prevalence of Neuropsychiatric Symptoms During Acute Crises in Persons With Dementia–A Systematic Review

**DOI:** 10.1097/WAD.0000000000000684

**Published:** 2025-08-12

**Authors:** Robert J. Brontsema, Maaike A. Pouw, Marike C. Schokker, Barbara C. van Munster, Hendrika J. Luijendijk

**Affiliations:** * Department of Primary and Long-Term Care, University Medical Center Groningen, Cure and Care in the Community Context (FOUR-C) Research Program; † University Medical Center Groningen, Alzheimer Center Groningen; ‡ Department of Geriatrics, Martini Hospital, Groningen, The Netherlands

**Keywords:** dementia, neuropsychiatric symptoms, delirium, review, crisis

## Abstract

**Background:**

Dementia affects millions worldwide, with neuropsychiatric symptoms (NPS) impacting up to 90% of those affected. These symptoms can escalate to crises, which present significant challenges for patients, caregivers, and health care systems.

**Objective:**

This study aimed to determine the prevalence of NPS and delirium in patients with dementia during acute crises.

**Methods:**

A comprehensive search was conducted across multiple bibliographic databases. Data extraction included NPS such as aggression and hallucinations, NPS measurement tool, delirium diagnoses, delirium screening tool, and discharge locations. Risk of bias was assessed using the Mixed Methods Appraisal Tool (MMAT), and a meta-analysis was performed. The review was reported following PRISMA guidelines.

**Results:**

Nineteen studies published from 1990 to 2023 were included. All were performed in hospitalized patients. Results indicated high heterogeneity in NPS prevalence: on medical wards, aggression 0.47 (95% CI: 0.35-0.59) was the most prevalent, followed by aggression/agitation 0.31 (95% CI: 0.15-0.48). In (gero)psychiatric wards, irritability 0.67 (95% CI: 0.52-0.79), and agitation 0.66 (95% CI: 0.59-0.73) were among the most common symptoms. In addition, 9 studies reported the rate of delirium; the pooled prevalence was 0.24 (95% CI: 0.11-0.38), with studies utilizing a screening tool reporting higher rates.

**Conclusion:**

These findings highlight the prevalence of NPS during crisis hospitalizations and the critical role of deliberate delirium screening. They enhance understanding of dementia crisis phenomenology and offer a foundation for targeted interventions to improve care quality. More research about crises in patients with dementia at home need to be performed.

In 2023 ∼55 million people are living with dementia worldwide, with around 10 million new cases every year.^1^ While dementia is often characterized by cognitive decline, the Behavioral and Psychological Symptoms of Dementia (BPSD) affect up to 90% of Persons with Dementia (PwD) over the course of the disease.^2^ These symptoms are not only harmful for the quality of life for the PwD,^3^ but could exceed the impact of cognitive symptoms on caregiver staff burden.^4^ BPSD, henceforth referred to as Neuropsychiatric Symptoms (NPS), are among the earliest signs of neurocognitive decline, and their severity increases over the course of the disease.^5^ NPS include delusions, hallucinations, agitation/aggression, depression, anxiety, euphoria, apathy, disinhibition, irritability, aberrant motor behavior, night-time behaviors, and appetite disorders.^6^

It is not uncommon for PwD to experience a crisis due to severe NPS. A crisis is defined by MacNeil Vroomen, Bosmans^7^ as “a process where there is a stressor(s) that causes an imbalance requiring an immediate decision which leads to a desired outcome and therefore crisis resolution. If the crisis is not resolved, the cycle continues.” Examples of such crises in PwD include malnutrition, or caregiver exhaustion. The causes of a crisis in PwD can be divided into 3 different categories. A crisis can have a somatic cause, like, for example, a hip-fracture; it can have a social cause, like a conflict with family members about whether the PwD should continue to drive; or a behavioral cause, due to NPS. Distinguishing between NPS caused by dementia and those stemming from delirium is often challenging due to overlapping symptoms. The differentiation is critical, as delirium is often reversible with timely identification and treatment. Failure to recognize delirium also increases the risk of accelerated cognitive decline and higher mortality.^8^

A systematic review on NPS during a crisis of PwD found that behavioral crises most often happen in the severe stages of dementia.^9^ During these crises, aggression and agitation were the most frequently reported behaviors, followed by delusions, wandering/absconding, and hallucinations. Furthermore, the authors suggest that crises are often not fully resolved in the long term, with patients being readmitted within a short timeframe. As the number of PwD are expected to increase,^1^ and workforce capacity is expected to decrease,^10^ the frequency of crisis situations can be expected to rise rapidly within the next few decades. Gaining insight into NPS displayed during these crises is paramount. The previous review about NPS in PwD during crisis was based on data published until June 20th, 2016.^9^ To our knowledge, no other review on this topic has been published since, although new studies were published. The current review had the following objectives:

1. To examine the prevalence of NPS and delirium during crises in PwD.
2. To examine the underlying causes of crises in PwD, categorized as behavioral, social, somatic, and other causes.
3. To investigate the outcome for PwD after a crisis.

## METHODS

The systematic review was reported following the Preferred Reporting Items for Systematic Reviews and Meta-Analyses (PRISMA) recommendations.^11^ The protocol was registered on Prospero (ID: 475825).

### Search Strategy and Eligibility

We searched the libraries of MEDLINE (through PubMed), Embase, Web of Science, PsychINFO, CINAHL, and the Cochrane Library. In addition, hand searches were performed of relevant reviews. All literature available up to August 8, 2023, was initially included, and a rerun of the search was conducted on October 23, 2024. The search strategy included strings covering 3 areas: dementia, crises, and behaviors. The complete list of strings can be found in Appendix 1, Supplemental Digital Content 1, http://links.lww.com/WAD/A533. The study placed no limitations on publication year, language, or publication status. Where required, authors were contacted to allocate full texts or clarify datapoints.

The articles were included if: (1) the study enrolled participants who had a diagnosis of any type of dementia. Studies involving a subset of participants with dementia were included if the data related to the subset could be isolated from the whole sample; (2) the study concerned a “crisis” or synonym in accordance to the definition of MacNeil Vroomen, Bosmans,^7^ and transpired in an acute or emergency situation; and (3) the study contained a description of NPS during a crisis as defined by the authors. With respect to criterion 2, the initial intent was to include all studies that concerned a crisis as defined by MacNeil Vroomen, Bosmans.^7^ However, during the literature selection process it became evident that using this definition encompassed a wide range of different situations (eg, planned admission, acute admission, and requiring a change in medication). Therefore, we refined the inclusion criterion to explicitly specify crises that gave rise to an acute or emergency situation.

Articles were excluded based on the following criteria: (1) studies that solely described the management of a crisis; (2) studies concerning crises that developed during inpatient stay; (3) case studies or opinion pieces; and (4) studies concerning end-of-life care.

### Selection Process

Two authors independently performed the initial screening based on titles and abstracts (R.J.B. and M.A.P.). Articles selected by both authors were immediately included. Instances where only one author had selected an article were re-evaluated and included if consensus was met. Disagreements were discussed and resolved by a third person (H.J.L.) where necessary. The screening of titles and abstracts was performed with the assistance of the machine learning algorithm ASReview,^12^ which ranked articles based on their textual similarities to previously selected articles, reducing time expenditure in the early screening phase. Screening was completed after identifying 100 consecutive irrelevant articles, which provides near 100% accuracy.^13^ If the full-text article was not readily accessible or lacked essential data, attempts were made to contact the authors for the required information.

### Data Extraction

The following data was extracted: (1) study characteristics (authors, setting, sample size, and study aim); (2) patient characteristics including medical history (age, sex, marital status, socioeconomic status, living situation before the crisis, type of dementia, history of psychiatric disease, social network size, and ethnicity); (3) NPS including the number of participants with delusions, hallucinations, confusion, attention disorder, aggression, wandering, stalking, anxiety, agitation, depression, apathy, disinhibition, self-neglect, sleep disorder, vocalizations, resisting care, euphoria, appetite change, irritability; (4) delirium screening tool/criteria, and number of delirium diagnoses; (5) the cause of the crisis: somatic, behavioral or social; and (6) living situation after the crisis. Two authors extracted the data independently from each included study (R.J.B. and M.A.P.). Disagreements between authors were discussed until consensus was reached.

### Risk of Bias Assessment

Two authors assessed the risk of bias independently (R.J.B. and M.A.P.) using the revised Mixed Methods Appraisal Tool (MMAT) version 2018.^14^ The MMAT assesses 5 core quality criteria for 5 different study designs (qualitative studies, randomized controlled trials, nonrandomized studies, quantitative studies, and mixed methods studies). We initially selected this tool with the expectation of encountering studies of various designs. However, eventually, only cross-sectional observational studies were included. Therefore, only the criteria for “quantitative studies” were utilized. Discrepancies in appraisal were resolved through discussion.

### Data Synthesis

Initially, we planned to synthesize the findings using a narrative approach in line with the “Guidance on the Conduct of Narrative Synthesis in Systematic Reviews” to construct a descriptive synthesis of the data if substantial heterogeneity was present (*I*² ≥50%) (Popay, Roberts).^15^ However, we found that the included studies described sufficiently homogenous designs to justify aggregating the prevalence rates. Specifically, all studies performed baseline measurements upon admission to a hospital ward. Nevertheless, the settings were found to vary (medical versus geropsychiatric/ psychogeriatric wards). Hence, we decided to conducted meta-analyses to pool the frequencies of individual NPS across these settings. To avoid exclusions due to a prevalence of zero, a continuity correction of 0.5 was applied. Statistical heterogeneity would be tested using the *I*² test. We used random effects models to take the heterogeneity into account.

The items of the NPI have been used as the overarching categories, given that the NPI was the most frequently used tool (n=5). When other tools had been used to measure symptoms covering a similar overarching concept (eg, day/night disturbance on BehaveAD and night-time behaviors on NPI) the data were pooled. Also, agitation and aggression symptoms were often reported as separate categories, and these items have been retained in addition to the NPI item agitation/aggression. Data based on tools that combined different types of NPS have been dismissed (eg, the item Hallucinations-Delusions from Rabins and Nicholson^16^).

In addition, we pooled the prevalence of delirium, the causes of crises, and discharge location across studies using a random-effect model. A sensitivity analysis was performed to assess heterogeneity between studies with and without the use of an NPS screening tool. Finally, studies lacking NPS prevalence data on admission were summarized narratively.

## RESULTS

### Study Search and Selection

**FIGURE 1:**
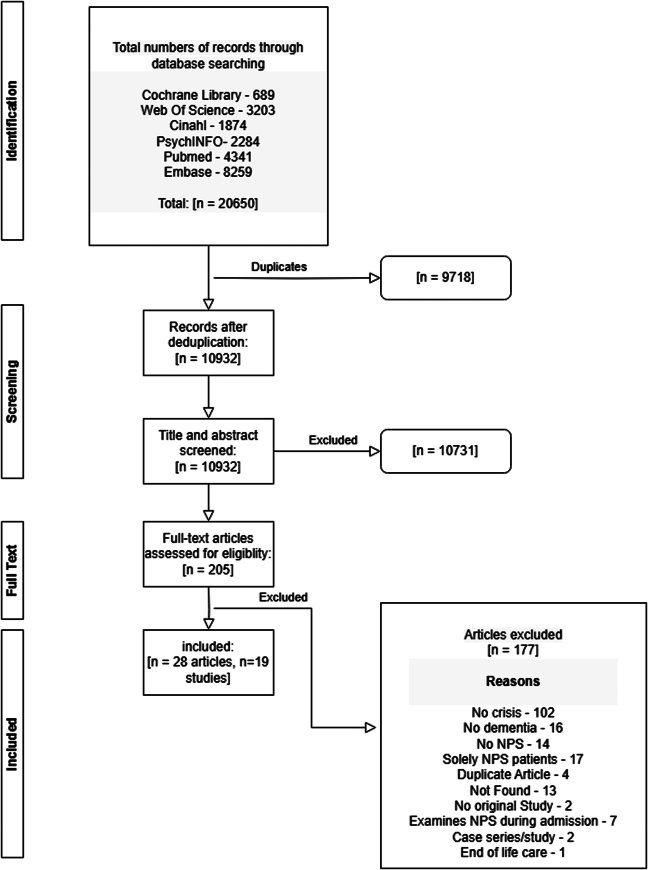
Flow diagram of the study selection process following PRISMA guidelines. Displays the number of records identified, screened, assessed for eligibility, and included in the final analysis, along with reasons for exclusions at each stage.

The initial search identified a total of 20,650 records. After deduplication, 10,932 records were assessed for eligibility by means of title/abstract screening. Then, 205 records were examined by means of a full-text assessment. A total of 28 papers covering 19 studies were included in the review (Fig. 1).

Table 1 presents the characteristics of the included studies. These studies spanned a wide range of publication years (1990 to 2022) and involved countries in Europe, North America, Australia, and Southeast Asia. The study designs included chart reviews, controlled trials and prospective cohort studies, of which only baseline data was used. Most studies were conducted in medical, surgical, geriatric, or mixed wards (henceforth referred to as “medical”), and a smaller number in (gero)psychiatric or psychogeriatric units (referred to as “psychiatric”).

**TABLE 1:** Study Characteristics

Author	Country	Type ward	Study design	Sample size	Dementia type	Age, mean	Female, n (%)	NPS tool
Moak 1990^26^	USA	Treatment Service	Prospective cohort	36	Mix	74.2	12 (33)	NA
Rabins 1991^16^	USA	Psychiatric unit	Retrospective chart review	121	NA	73	68 (56)	NA
Neville 1999^25^	UK	General Hospital	Cross-sectional questionnaire	230	Mix	79.9	131 (57)	Tick-box type questionnaire
Marengoni 2004^32^	Italy	Geriatric unit	Retrospective chart review	148	Mix	82.3	107 (72)	NA
Takacs 2004^31^	Hungary	Psychiatric unit	Retrospective survey	79	Mix	77.7	54 (68)	NA
Tan 2005* ^20^	Singapore	Psychogeriatric ward	Cross-sectional	42	Mix	76.1	20 (48)	NPI-NH
Soto 2008 and 2012^21^	France	Internal medicine and geriatrics	Prospective observational	568	Mix	81	305 (54)	NA
Nourhashemi 2001^24^	France	Internal medicine and geriatrics	Prospective cohort	118	Mix	82	85 (72)	NA
Sampson 2014 and 2015^23^; Kupeli 2017; White 2017^23^	UK	Medical acute admissions ward	Prospective cohort	230	Mix	87.2	151 (66)	Behave-AD
Silwanowicz 2016^22^	USA	Mixed (medical+psychiatric)	Retrospective cohort	156	NA	80	93 (60)	NPI-Q
Pitkanen 2018^18^; Alanen 2015	Finland	(Psycho)geriatric ward	Retrospective cohort	175	Mix	77.8	96 (55)	NPI
Chenoweth 2022† ^33^	Australia	Aged care and surgical/medical	Nonrandomized trial	47	NA	77	16 (34)	CMAI
Jones 2022^34^	Canada	Emergency department	Retrospective cohort	175,863	AD	82‡	101,649 (58)	NA
Drazich 2022§ ^28^; Resnick 2023	USA	General medical unit	Cross-sectional Descriptive	294	NA	83.2	187 (64)	(Assumed∥) NPI
Boltz 2023¶ ^17^; Berish 2024	USA	Medical and medical/surgical	Cohort embedded in trial	455	NA	81.5	269 (59)	NPI-Q
Lagarto 2018^29^; Esteves 2016	Portugal	Male geriatric ward	Prospective cohort	270	NA	83.6	0	NPI
Tunis 2002^27^	USA	(Gero)psychiatric ward	Retrospective observational	2256	Mix	81	1482 (66)	PGDRS
Pitkala 2004^30^	Finland	Geriatric ward	Cross-sectional	95	NA	NA	NA	NA
Sinvani 2023^19^	USA	Medical unit	Prospective feasibility trial	158	NA	85.2	100 (63)	NPI-Q

* This sample excludes participants with any serious medical condition.

† This sample excludes participant with a delirium, or unstable physical of psychiatric illness.

‡ Median.

§ This sample excludes participants with a major acute psychiatric disorder affecting cognition.

∥ We assumed usage of the NPI due to the items being presented as a complete match with the items presented in the NPI.

¶ This sample excludes patients with any significant neurological condition associated with cognition or major psychiatric disorders.

AD stands for Alzheimer disease, NA for not applicable (data were based on symptoms registered in the medical file).

Table 2 shows the results of the risk of bias assessment, which highlight variability across studies in key domains of bias. Notable weaknesses were found in terms of representativeness and sampling. Representativeness was often affected by the type or number of exclusion criteria. Weaknesses in measurements were primarily due to not using a specified tool for NPS, or not reporting the prevalence of NPS at all.

**TABLE 2:**
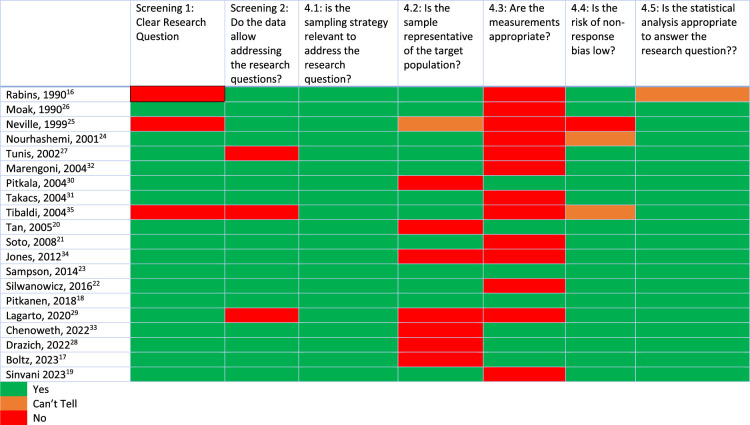
Mixed Method Assessment Tool (MMAT) Scores

Most studies reported basic demographics of the study population (Table 1). The proportion of females in individual studies ranged from 22% to 72%. The mean ages ranging from 73 to 87.2 years and the weighted average age was 81.1 years (SD=2.4). Marital status data were reported in only 3 studies^17–19^ indicating that 34% to 47% of participants were married. Nine studies reported that the proportion of participants living at home ranged from 33% to 69%.^16,18,20–26^ Eight reported the background of the PwD,^16,17,19,20,22,23,27,28^ 2 mentioned some aspect of socioeconomic-status (SES),^17,29^ 2 discussed previous prior psychiatric hospitalizations,^22,27^ and none of them discussed a potential small network of the PwD. The duration of hospital stay was provided in 5 studies,^16,18,21,25,26^ ranging from 10.7 to 84 days.

### Neuropsychiatric Symptoms and Delirium

**FIGURE 2:**
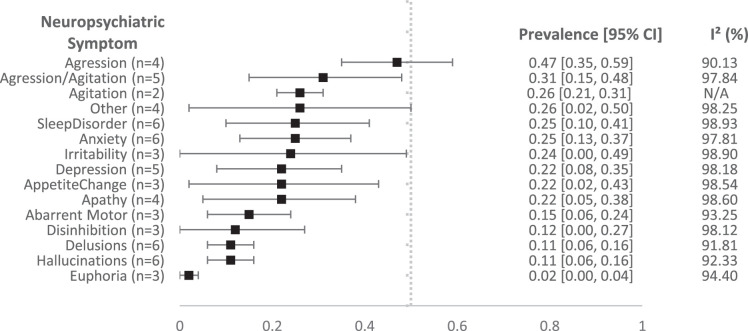
Forest plot showing the pooled prevalence of neuropsychiatric symptoms among patients on acute medical wards. Each symptom is represented with its point estimate and 95% CI and an *I* ^2^ score.

Twelve studies reported data that could be pooled. To decrease heterogeneity, we stratified the pooled prevalences by type of ward: medical wards^17,22–26,28–30^ versus psychiatric wards.^16,20,31^ Figure 2 shows the pooled proportion of PwD with NPS at the time of an acute crisis admitted on a medical ward.^17,21–23,25,26,28,30^ The study of Marengoni et al^32^ was excluded from this analysis due to solely providing the prevalence of NPS in patients whose primary cause of admission was behavioral in nature. The pooled proportion of PwD with aggression was the highest at 0.47 (95% CI: 0.35-0.59). The second highest was that of aggression/ agitation (combined) at 0.31 (95% CI: 0.15-0.48), followed by agitation at 0.26 (95% CI: 0.21-0.31) and sleep disorder at 0.25 (95% CI: 0.10-0.41). The lowest prevalence was reported for euphoria at 0.02 (95% CI: 0.00-0.04), followed by hallucinations and delusions, both at 0.11 (95% CI: 0.06-0.16). Forest plots of the individual symptoms on the medical ward can be found in Appendix 2, Supplemental Digital Content 2, http://links.lww.com/WAD/A534.

**FIGURE 3:**
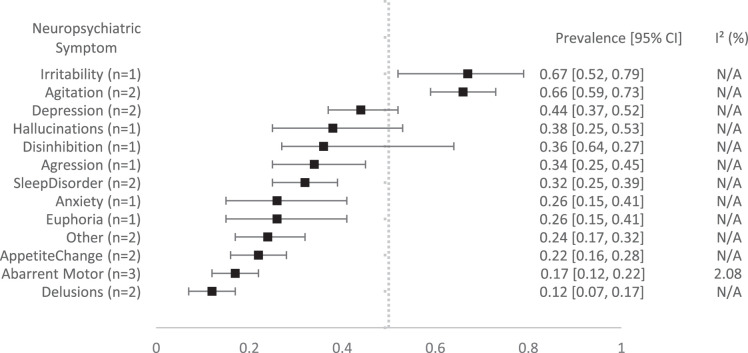
Forest plot showing the pooled prevalence of neuropsychiatric symptoms among patients on acute (gero)psychaitric wards. Each symptom is represented with its point estimate and 95% CI and an *I* ^2^ score.

As shown in Figure 3, the most prevalent NPS in patients admitted to a psychiatric ward were irritability [0.67 (95% CI: 0.52-0.79)], agitation [0.66 (95% CI: 0.59-0.73)], and depression [0.44 (95% CI: 0.37-0.52)]. The forest plots of the individual symptoms on the psychiatric wards can be found in Appendix 3, Supplemental Digital Content 3, http://links.lww.com/WAD/A535.

Six studies^18,19,27,29,33,34^ did not provide suitable NPS data for pooling, but reported comparable results. The study of Pitkänen, Alanen^18^ found aggression and agitation to be among the most severe NPS. The study of Tunis, Edell^27^ noted restless behaviors to be the most prevalent NPS, occurring in 65.1% of patients. Meanwhile, Chenoweth, Williams,^33^ found that at baseline the intervention group had a mean Cohen-Mansfield Agitation Index (CMAI) score of 16.08 (SD=5.71), compared with 20 (SD=6.57) in the control group. The feasibility trial by Sinvani and colleagues (2023) reported a mean NPI-Q score at baseline of 15.8 for the intervention group, and 10.5 for the control group, but did not differentiate by individual symptom.

Ten of 19 studies reported the prevalence of delirium, and showed a pooled prevalence of 0.26 (95% CI: 0.13-0.39). Five studies actively screened for delirium with the DSM-V, CAM, or CAM-S criteria.^17,19,23,29,30^ These studies had a pooled delirium rate of 0.38 (95% CI: 0.15-0.60). In contrast, the 5 studies that did not report using specified criteria or a tool showed a pooled rate of 0.14 (95% CI: 0.06-0.22).^16,22,25,31,32^

### Causes of Crises and Discharge Location

Fourteen studies reported data that could be pooled. Studies with the total number of causes exceeding the sample size were not pooled due to the inability to aggregate the data about causes of admission.^25^ Four studies concerning a psychiatric ward provided only data for one admission type.^16,18,20,31^

Five studies reported the prevalence of behavioral causes for crises on medical wards,^21,24,26,32,34^ and the pooled prevalence was 0.40 (95% CI: 0.11-0.68). The study of Takacs et al^31^ on a (gero)psychiatric ward showed a prevalence of behavioral cause for crisis of 0.85 (95% CI: 0.75-0.91). The pooled prevalence on medical wards of somatic causes was 0.63 (95% CI: 0.38-0.88) based on 6 studies,^21,23,24,30,34,35^ and that of social causes was 0.04 (95% CI: 0.03-0.06) based on 2 studies.^21,24^ The pooled prevalence of other reasons for acute admission, like treatment refusal and transfers from other hospitals, was 0.09 (95% CI: 0.05-0.13) and they were reported in 5 articles.^21,24,26,30,34^

Four studies reported on mortality rates during hospitalization^23,24,26,31^ resulting in a pooled proportion of 0.05 (95% CI: 0.00-0.11). Five studies also provided information on the prevalence of extended hospitalization following acute admission,^16,22,24,26,31^ showing a pooled proportion of 0.45 (95% CI: 0.16-0.74). Six studies reported the proportion of patients who were discharged to their homes after the crisis admission,^16,18,26,31,33,34^ resulting in a pooled proportion of 0.26 (95% CI: 0.11-0.41). Lastly, 5 studies reported on patients who were institutionalized at discharge,^16,18,26,31,33^ with a pooled proportion of 0.39 (95% CI: 0.23-0.55).

## DISCUSSION

This review covered 19 studies that investigated the prevalence of NPS in PwD during an acute crisis. The most prevalent symptoms in acute medical settings were aggression, aggression/agitation, agitation, and sleep disturbances, each showing a prevalence of 25% or higher, while less frequent symptoms like euphoria, hallucinations, and delusions were prevalent in up to 11%. PwD admitted to (psycho)geriatric wards showed overall higher prevalence rates of irritability, agitation, and depression. Delirium was observed in 26% of patients.

In line with the review of Backhouse, Camino et al,^9^ we found that agitation and aggression were the most reported behaviors at a time of acute crisis in PwD. These symptoms are deemed to be severely distressing and dangerous to patients and caregivers.^36^ There was, nevertheless, a high level of heterogeneity in the prevalence of NPS, despite being stratified by type of ward. This heterogeneity might be caused by a difference in the level of urgency of the admissions between studies, differences between countries, differences in participant eligibility criteria, or differences in the measurement of NPS. However, a sensitivity analysis dividing studies that did and did not use a specified tool showed minimal change in heterogeneity. In addition, current screening tools for NPS allow for a considerable degree of subjectivity.

The prevalence of NPS in (gero)psychiatric wards compared with medical wards appeared higher for conditions, such as irritability, agitation, and depression. This discrepancy is likely attributable to underlying psychiatric conditions, or the symptoms were the main reason for referring the PwD to such a ward. It is also important to note that the limited sample size of studies reporting prevalence data from (gero)psychiatric wards necessitates caution when interpreting these findings.

Due to the acute setting of the included studies in this review, a high prevalence of delirium may be anticipated, and we observed a pooled rate of 26%. Although data on the prevalence of delirium specifically in PwD are limited, research on general geriatric populations offers some context. In a review about delirium among geriatric medical inpatients, the prevalence ranged from 10% to 31% at the time of admission (Siddiqi, House, and Holmes).^37^ Our review found a delirium prevalence of 14% when no screening tool was reported, and 38% when a screening tool was utilized. This discrepancy could be explained by undiagnosed delirium due to a lack of specific screening, as highlighted in the study of Al Farsi, Al Alawi^38^ who found that 35.4% of delirium was undiagnosed at admission. This finding further highlights the importance of proper screening tools to be utilized, even though tool usage cannot be expected to remove all variation.^37^

Another possible explanation for the variation in prevalence rates lies in the availability of extensive primary care. In countries without comprehensive primary care, most patients with delirium symptoms may be referred directly to a hospital. However, early delirium detection before an acute admission may be preferable. In a Dutch study, 20% of patients who had been referred to a geropsychiatric department for outpatient dementia screening had delirium, and 80% of the PwD an emergency referral (Quispel-Aggenbach, Schep-de Ruiter).^39^ Most of these cases of delirium were managed successfully at home. Hence, early detection avoided the need for acute hospitalization and the risk of further disorientation as a result of it.

### Strengths and Limitations

The strengths of this review include the use of standardized procedures for data extraction from the included studies and the application of strict inclusion criteria to ensure accurate data aggregation. However, many of the included studies applied exclusion criteria, like the exclusion of PwD with delirium, that will have impacted the prevalence of NPS and delirium. The included studies also used different screening tools and varied in reporting practices, making it challenging to combine these populations in a meta-analysis. Use of a more standardized, objective NPS-screening tool could help reduce this variability. For example, employing a tool with clearly defined cutoff points or integrating sensor technology could further enhance consistency.

Furthermore, this study aimed to examine the prevalence of NPS in people with dementia during acute crises across various settings, including those living in their own homes, nursing homes, rehabilitation centers, and hospitals. However, most of the included studies were performed in medical hospital wards or emergency rooms, creating a knowledge gap for other environments. Also, after separating (gero)psychiatric wards from medical wards, the degree of heterogeneity remained high. As a result, caution is warranted when attempting to generalize the findings. In addition, while these data provide a broad overview of NPS prevalence in acute wards, they do not capture the intensity of these symptoms.

## CONCLUSIONS

This systematic review provides insight into the prevalence of neuropsychiatric symptoms in people with dementia acutely admitted to medical and psychiatric wards. Aggression and agitation emerged as the most prevalent symptoms in the medical wards, underscoring their distressing nature and need for acute care. The findings also highlighted the importance of using delirium screening tools in acute settings to increase detection rates of possible treatable causes of NPS. These findings emphasize the need for targeted interventions to recognize and manage NPS in an effective and safe manner, alongside standardized protocols to prevent conditions like delirium from going unrecognized. Such measures not only benefit PwD during hospitalization, but also support their well-being following discharge.

## Supplementary Material
