# Supplementary material for: The Prevalence of Neuropsychiatric Symptoms During Acute Crises in Persons With Dementia–A Systematic Review
Source: Alzheimer Dis Assoc Disord. 2025 Aug 12;39(4):342–9. doi: 10.1097/WAD.0000000000000684 (PMC12637143; doi:10.1097/WAD.0000000000000684)
Supplement: Supplementary file 1 [file wad-39-342-s001.docx]

**Appendix 1:** Searchterms

**Pubmed/Medline**

(Dementia[Mesh] OR Dement*[Tiab] OR Alzheimer*[Tiab] OR Frontotemporal lobar degeneration[Tiab] OR Lewy body d*[Tiab])

AND

(Emergenc*[Tiab] OR Crisis[Tiab] OR Crises[Tiab] OR Crisis intervention[Mesh] OR Hospitalization[Mesh:NoExp] OR Hospitali*[Tiab] OR Institutionali* OR Hospital admission*[Tiab] OR Patient Admission[Mesh] OR patient admission*[Tiab] OR Acute admission*[Tiab] OR Nursing home admission*[Tiab] OR Emergency medical services[Mesh])

AND

(Behavioral symptoms[Mesh] OR Delir*[Tiab] OR Sepsis-associated encephalopathy[Tiab] OR Psychotic[Tiab] OR Psychos*[Tiab] OR Obsess*[tiab] OR Delusion*[Tiab] OR Hallucinat*[Tiab] OR Confus*[Tiab] OR Behav*[Tiab] OR Affective*[Tiab] OR Aggress*[Tiab] OR Bullying[Tiab] OR Stalking[Tiab] OR Self-mutilat*[Tiab] OR self-injur*[Tiab] OR Wandering[Tiab] OR Agit*[Tiab] OR Apath*[Tiab] OR Anxi*[Tiab] OR Disinhibit*[Tiab])

NOT

(Casestud*[Tiab] OR Case Reports[Publication Type] OR Case report*[Tiab])

**Psychinfo**

(MM "Dementia" OR MM "AIDS Dementia Complex" OR MM "Alzheimer's Disease" OR MM "Dementia with Lewy Bodies" OR MM "Frontotemporal Lobar Degeneration" OR MM "Presenile Dementia" OR MM "Senile Dementia" OR MM "Vascular Dementia" OR TI(Dement* OR Alzheimer* OR "frontotemporal lobar degeneration" OR "Lewy Body*") OR AB (Dement* OR Alzheimer OR "frontotemporal lobar degeneration" OR "Lewy Body d*") OR KW (Dement* OR Alzheimer OR "frontotemporal lobar degeneration" OR "Lewy Body d*"))

AND

(MM "Crisis Intervention" OR MM "Hospitalization" OR TI(Emergenc* OR Crisis OR Crises OR Hospitali* OR Institutionali* OR "Hospital admission*" OR "Patient admission*" OR "Acute admission*" OR "nursing home admission*" OR “Emergency medical services”) OR AB (Emergenc* OR Crisis OR Crises OR Hospitali* OR Institutionali* OR "Hospital admission*" OR "Patient admission*" OR "Acute admission*" OR "nursing home admission*" OR “Emergency medical services”) OR KW (Emergenc* OR Crisis OR Crises OR Hospitali* OR Institutionali* OR "Hospital admission*" OR "Patient admission*" OR "Acute admission*" OR "nursing home admission*" OR “Emergency medical services”)))

AND

(TI("Behavio*ral symptoms" OR Deliri* OR "Sepsis-associated encephalopathy" OR Psychotic OR Psychos* OR Obsess* OR Delusion* OR Hallucinat* OR Confus* OR Behav* OR Affective* OR Aggress* OR Bullying OR Stalking OR "Self mutilat *" OR "Self injur*" OR Wandering OR Agit* OR Apath* OR Anxi* OR Disinhibit*) OR AB ("Behavio*ral symptoms" OR Deliri* OR "Sepsis-associated encephalopathy" OR Psychotic OR Psychos* OR Obsess* OR Delusion* OR Hallucinat* OR Confus* OR Behav* OR Affective* OR Aggress* OR Bullying OR Stalking OR "Self mutilat *" OR "Self injur*" OR Wandering OR Agit* OR Apath* OR Anxi* OR Disinhibit*) OR KW ("Behavio*ral symptoms" OR Deliri* OR "Sepsis-associated encephalopathy" OR Psychotic OR Psychos* OR Obsess* OR Delusion* OR Hallucinat* OR Confus* OR Behav* OR Affective* OR Aggress* OR Bullying OR Stalking OR "Self mutilat *" OR "Self injur*" OR Wandering OR Agit* OR Apath* OR Anxi* OR Disinhibit*))

OR "Self mutilat*" OR "Self injur*" OR Wandering OR Agit* OR Apath* OR Anxi* OR Disinhibit*))

NOT

(TI("Case stud*" OR "Case report") OR AB("Case stud*" OR "Case report") OR KW("Case stud*" OR "Case report"))

**Embase**

(‘Dementia’/exp OR Dement*:ti,ab,kw OR Alzheimer*:ti,ab,kw OR 'Frontotemporal lobar degeneration':ti,ab,kw OR 'Lewy body d*':ti,ab,kw)

AND

(Emergenc*:ti,ab,kw OR Crisis:ti,ab,kw OR Crises:ti,ab,kw OR 'Crisis intervention'/exp OR ‘Hospitalization’/exp OR Hospitali*:ti,ab,kw OR Institutionali*:ti,ab,kw OR 'Hospital admission'/exp OR 'Hospital admission*':ti,ab,kw OR 'Patient admission*':ti,ab,kw OR 'Acute admission*':ti,ab,kw OR 'Nursing home admission*':ti,ab,kw)

AND

('Behavio*ral symptom*':ti,ab,kw OR Delirium:ti,ab,kw OR 'Sepsis-associated encephalopathy':ti,ab,kw OR Psychotic:ti,ab,kw OR Psychos*:ti,ab,kw OR Obsess*:ti,ab,kw OR Delusion*:ti,ab,kw OR Hallucinat*:ti,ab,kw OR Confus:ti,ab,kw OR Behav*:ti,ab,kw OR Affective*:ti,ab,kw OR Aggress*:ti,ab,kw OR Bullying:ti,ab,kw OR Stalking:ti,ab,kw OR 'self mutilat*':ti,ab,kw OR ‘Self Injur*’:ti,ab,kw OR Wandering:ti,ab,kw OR Agit*:ti,ab,kw OR Apath*:ti,ab,kw OR Anxi*:ti,ab,kw OR Disinhibit*:ti,ab,kw)

NOT

('Case stud*':ti,ab,kw OR 'Case report':ti,ab,kw)

**Web of Science**

(TI=((Dement* OR Alzheimer* OR "frontotemporal lobar degeneration" OR "Lewy Body d*")) OR AB=((Dement* OR Alzheimer* OR "frontotemporal lobar degeneration" OR "Lewy Body d*")) OR AK=((Dement* OR Alzheimer* OR "frontotemporal lobar degeneration" OR "Lewy Body d*")))

AND

(TI=(("Behavi*oral symptom*” OR Deliri* OR "Sepsis-associated encephalopathy" OR Psychotic OR Psychos* OR Obsess* OR Delusion* OR Hallucinat* OR Confus* OR Behav* OR Affective* OR Aggress* OR Bullying OR Stalking OR "Self mutilat*" OR "Self injur*" OR Wandering OR Agit* OR Apath* OR Anxi* OR Disinhibit*) )

OR AB=(("Behavi*oral symptom*” OR Deliri* OR "Sepsis-associated encephalopathy" OR Psychotic OR Psychos* OR Obsess* OR Delusion* OR Hallucinat* OR Confus* OR Behav* OR Affective* OR Aggress* OR Bullying OR Stalking OR "Self mutilat*" OR "Self injur*" OR Wandering OR Agit* OR Apath* OR Anxi* OR Disinhibit*))

OR AK=(("Behavi*oral symptom*” OR Deliri* OR "Sepsis-associated encephalopathy" OR Psychotic OR Psychos* OR Obsess* OR Delusion* OR Hallucinat* OR Confus* OR Behav* OR Affective* OR Aggress* OR Bullying OR Stalking OR "Self mutilat *" OR "Self injur*" OR Wandering OR Agit* OR Apath* OR Anxi* OR Disinhibit*) ))

AND

(TI=((Emergenc* OR Crisis OR Crises OR "Crisis Intervention*" OR Hospitali* OR Institutionali* OR "Hospital admission*" OR "Patient admission*" OR "Acute admission*" OR "nursing home admission*" OR “Emergency medical services”))

OR AB=((Emergenc* OR Crisis OR Crises OR "Crisis Intervention*" OR Hospitali* OR Institutionali* OR "Hospital admission*" OR "Patient admission*" OR "Acute admission*" OR "nursing home admission*" OR “Emergency medical services”))

OR AK=((Emergenc* OR Crisis OR Crises OR "Crisis Intervention*" OR Hospitali* OR Institutionali* OR "Hospital admission*" OR "Patient admission*" OR "Acute admission*" OR "nursing home admission*" OR “Emergency medical services”)))

NOT (TI=(("Case stud*" OR "Case report")) OR AB=(("Case stud*" OR "Case report")) OR AK=(("Case stud*" OR "Case report")))

**Cinahl**

(MH "Dementia" OR TI(Dement* OR Alzheimer* OR "frontotemporal lobar degeneration" OR "Lewy Body*") OR AB (Dement* OR Alzheimer* OR "frontotemporal lobar degeneration" OR "Lewy Body d*"))

AND

(MH "Crisis Intervention" OR MH "Hospitalization" OR MH "Emergency Medical Services" OR TI(Emergenc* OR Crisis OR Crises OR Hospitali* OR Institutionali* OR "Hospital admission*" OR "Patient admission*" OR "Acute admission*" OR "nursing home admission*") OR AB (Emergenc* OR Crisis OR Crises OR Hospitali* OR Institutionali* OR "Hospital admission*" OR "Patient admission*" OR "Acute admission*" OR "nursing home admission*"))

AND

(MH "Behavioral Symptoms" OR TI(Deliri* OR "Sepsis-associated encephalopathy" OR Psychotic OR Psychos* OR Obsess* OR Delusion* OR Hallucinat* OR Confus* OR Behav* OR Affective* OR Aggress* OR Bullying OR Stalking OR "Self mutilat *" OR "Self injur*" OR Wandering OR Agit* OR Apath* OR Anxi* OR Disinhibit*) OR AB(Deliri* OR "Sepsis-associated encephalopathy" OR Psychotic OR Psychos* OR Obsess* OR Delusion* OR Hallucinat* OR Confus* OR Behav* OR Affective* OR Aggress* OR Bullying OR Stalking OR "Self mutilat *" OR "Self injur*" OR Wandering OR Agit* OR Apath* OR Anxi* OR Disinhibit*))

NOT

(TI("Case stud*" OR "Case report") OR AB("Case stud*" OR "Case report"))

**Cochrane**

ID Search

#1 MeSH descriptor: [Dementia] explode all trees

#2 (Dement*):ti,ab,kw

#3 (Alzheimer*):ti,ab,kw

#4 (Frontotemporal NEXT lobar NEXT degeneration*):ti,ab,kw

#5 (Lewy NEXT body NEXT disease*):ti,ab,kw

#6 #1 OR #2 OR #3 OR #4 OR #5

#7 MeSH descriptor: [Emergencies] this term only

#8 MeSH descriptor: [Crisis Intervention] explode all trees

#9 MeSH descriptor: [Hospitalization] this term only

#10 MeSH descriptor: [Patient Admission] explode all trees

#11 MeSH descriptor: [Emergency Medical Services] explode all trees

#12 (Emergenc*):ti,ab,kw

#13 (Crisis):ti,ab,kw

#14 (Crises):ti,ab,kw

#15 (Hospital NEXT admission*):ti,ab,kw

#16 (Institutionali*):ti,ab,kw

#17 (Patient NEXT admission*):ti,ab,kw

#18 (Acute NEXT admission*):ti,ab,kw

#19 (Nursing NEXT home NEXT admission*):ti,ab,kw

#20 #7 OR #8 OR #9 OR #10 OR #11 OR #12 OR #13 #14 OR #15 OR #16 OR #17 OR #18 OR #19

#21 MeSH descriptor: [Behavioral Symptoms] explode all trees

#22 (Delir*):ti,ab,kw

#23 (Sepsis NEXT Associated NEXT encephalopathy):ti,ab,kw

#24 (Psychotic):ti,ab,kw

#25 (psychos*):ti,ab,kw

#26 (Obsess*):ti,ab,kw

#27 (Delusion*):ti,ab,kw

#28 (Hallucinat*):ti,ab,kw

#29 (Confus*):ti,ab,kw

#30 (Behav*):ti,ab,kw

#31 (Affective*):ti,ab,kw

#32 (Agress*):ti,ab,kw

#33 (Bullying):ti,ab,kw

#34 (Stalking):ti,ab,kw

#35 (Self-mutil*:ti,ab,kw)

#36 (Self-injur*):ti,ab,kw

#37 (Wandering):ti,ab,kw

#38 (Agit*):ti,ab,kw

#39 (apath*):ti,ab,kw

#40 (Anxi*):ti,ab,kw

#41 (Disinhibit*):ti,ab,kw

#42 #21 OR #22 OR #23 OR #24 OR #25 OR #26 OR #27 OR #28 OR #29 OR #30 OR #31 OR #32 OR #33 OR #34 OR #35 OR #36 OR #37 OR #38 OR #39 OR #40 OR #41

#43 (Casestud*):ti,ab,kw

#44 (Case report*):ti,ab,kw

#45 #43 OR #44

#46 #6 AND #20 AND #42 NOT #45
