# Supplementary material for: The Prevalence of Neuropsychiatric Symptoms During Acute Crises in Persons With Dementia–A Systematic Review
Source: Alzheimer Dis Assoc Disord. 2025 Aug 12;39(4):342–9. doi: 10.1097/WAD.0000000000000684 (PMC12637143; doi:10.1097/WAD.0000000000000684)
Supplement: Supplementary file 2 [file wad-39-342-s002.docx]

**Appendix 2:** Prevalence individual symptoms Medical Ward

**Appendix 3:** Prevalence individual symptoms gero(psychiatric) ward
